# STAT3 ameliorates cognitive deficits via regulation of NMDAR expression in an Alzheimer's disease animal model

**DOI:** 10.7150/thno.56541

**Published:** 2021-03-13

**Authors:** Hua-Li Wan, Xiao-Yue Hong, Zai-Hua Zhao, Ting Li, Bing-Ge Zhang, Qian Liu, Qun Wang, Shi Zhao, Jian-Zhi Wang, Xue-Feng Shen, Gong-Ping Liu

**Affiliations:** 1 Department of Pathophysiology, School of Basic Medicine and the Collaborative Innovation Center for Brain Science, Key Laboratory of Ministry of Education of China and Hubei Province for Neurological Disorders, Tongji Medical College, Huazhong University of Science and Technology, Wuhan 430030, China.; 2 Department of Occupational and Environmental Health and the Ministry of Education Key Lab of Hazard Assessment and Control in Special Operational Environment, School of Public Health, Fourth Military Medical University, No 169 of West Changle Road, Xi'an, Shaanxi, 710032, China.; 3 Department of Endocrinology, Wuhan Central Hospital, 26 Shengli Street, Jiang'an District, Wuhan 430014, China.; 4 Co-innovation Center of Neuroregeneration, Nantong University, Nantong, JS 226001, China.

**Keywords:** Tau, STAT3, synapse, memory deficit, NMDAR

## Abstract

**Background:** Abnormal tau accumulation in the brain has a positively correlation with neurodegeneration and memory deterioration, but the mechanism underlying tau-associated synaptic and cognitive impairments remains unclear. Our previous work has found that human full length tau (hTau) accumulation activated signal transducer and activator of transcription-1 (STAT1) to suppress N-methyl-D-aspartate receptors (NMDARs) expression, followed by memory deficits. STAT3 also belongs to STAT protein family and is reported to involve in regulation of synaptic plasticity and cognition. Here, we investigated the role of STAT3 in the cognitive deficits induced by hTau accumulation.

**Methods:** *In vitro* studies HEK293 cells were used. EMSA, Luciferase reporter assay, and Immunoprecipitation were applied to detect STAT3 activity. *In vivo* studies, AAV virus were injected into the hippocampal CA3 region of C57 mice. Western blotting, quantitative real-time polymerase chain reaction, and immunofluorescence were applied to examine the level of synaptic proteins. Electrophysiological analysis, behavioral testing and Golgi impregnation were used to determine synaptic plasticity and memory ability recovery after overexpressing STAT3 or non-acetylated STAT1.

**Results:** Our results showed that hTau accumulation acetylated STAT1 to retain STAT3 in the cytoplasm by increasing the binding of STAT1 with STAT3, and thus inactivated STAT3. Overexpressing STAT3 or non-acetylated STAT1 ameliorated hTau-induced synaptic loss and memory deficits by increasing the expression of NMDARs.

**Conclusions:** Taken together, our study indicates that hTau accumulation impaired synaptic plasticity through STAT3 inactivation induced suppression of NMDARs expression, revealing a novel mechanism for hTau-associated synapse and memory deficits.

## Introduction

The mammalian signal transducer and activator of transcription (STAT), consisted of seven members, i.e., STAT1, STAT2, STAT3 and STAT4, STAT5a, STAT5b and STAT6, are latent self-signaling transcription factors in cytoplasm, and rapidly turn on gene expression in nuclei used by most cytokine receptors 1. Among STAT family, STAT1, 3, 5 and 6 are differentially expressed in the brain 2. STATs share structurally and functionally conserved domains, including the amino-terminal domain, the coiled-coiled domain, the DNA binding domain, the linker domain and the SH2/tyrosine activation domain. The carboxy-terminal transcriptional activation domain contributing to STAT specificity, is quite divergent 3. As the most highly conserved STAT domain, SH2 domains play an important role in signaling through their capacity to bind to specific phosphotyrosine motifs of cytokine or growth factor receptors 1. STATs are critical mediators of functional responses and specificity in cytokine signaling. As transcription factors, STATs activity is regulated by its phosphorylation 2, 3, for example, phosphorylation of STAT1 at Tyr701 or phosphorylated STAT3 at Tyr705 stimulates STAT1 or STAT3 dimerization, nuclear translocation, DNA binding, and activation of target genes transcription 1-6.

It's well known that STATs play a role in development, cell growth and differentiation, proliferation, immune responses, cell survival and apoptosis 5-12. Recently, STAT1 and STAT3 have been reported to involve in regulation of synaptic plasticity and cognition 13-17. The JAK/STAT pathway plays an essential role in the induction of NMDA-receptor dependent long-term depression in the hippocampus 18. STAT1 negatively regulated spatial memory formation and mediates the memory-impairing effect of ChAT by downregulation of NMDARs expression via transcription factor LB1 16. STAT3 is reported to localize in the mitochondria and modulate the activity of the electron transport chain. Pharmacological inhibition of the JAK2/STAT3 axis not only induced significant loss of spatial working memory by downregulating an acetylcholine-producing enzyme choline acetyltransferase but also desensitized the M1-type muscarinic acetylcholine receptor 19. Amyloid-β caused memory impairment by disturbing the JAK2/STAT3 axis in hippocampal neurons 20.

Recently, we found that STAT1 negative regulated NMDARs mRNA and protein levels by directly binding the gamma interferon activation site (GAS) elements in the promoter of NMDARs DNA sequence, human wild type full length microtubule associated protein tau (hTau) accumulation activated JAK2 to phosphorylate STAT1 at Tyr701 and promote STAT1 to translocate into the nucleus and negatively regulate NMDARs expression 21. Like STAT1, STAT3 binds to a similar GAS element of target genes and also involved in cognition regulation 8, 9, and phospho-STAT3 level in hippocampal neurons age-dependently decreased in both AD model mice and AD patients 20, we speculate that STAT3 may also play a key role in the hTau-induced cognitive deficits.

Using AAV-hTau injected into the hippocampus of 2-m-old C57 mice to mimic intraneuronal tau accumulation as seen in the majority of AD cases, we found that overexpression of hTau acetylated STAT1 to increase STAT1 binding with STAT3 in the cytoplasmic fraction, and then, decreased STAT3 translocation into the nucleus and inactivated STAT3, though phosphorylated level of STAT3 at Tyr705 increased. Further study found that overexpressing STAT3 or non-acetylated STAT1 (STAT1KR) ameliorated the hTau-induced synaptic plasticity impairment and cognitive deficits with upregulated NMDARs expression. This study reveals a new mechanism underlying hTau accumulation induced cognitive deficits, and provides acetylated STAT1 or wild type STAT3 as a new therapy target for tauopathies.

## Results

### Intracellular hTau accumulation inactivates STAT3

**Figure 1:**
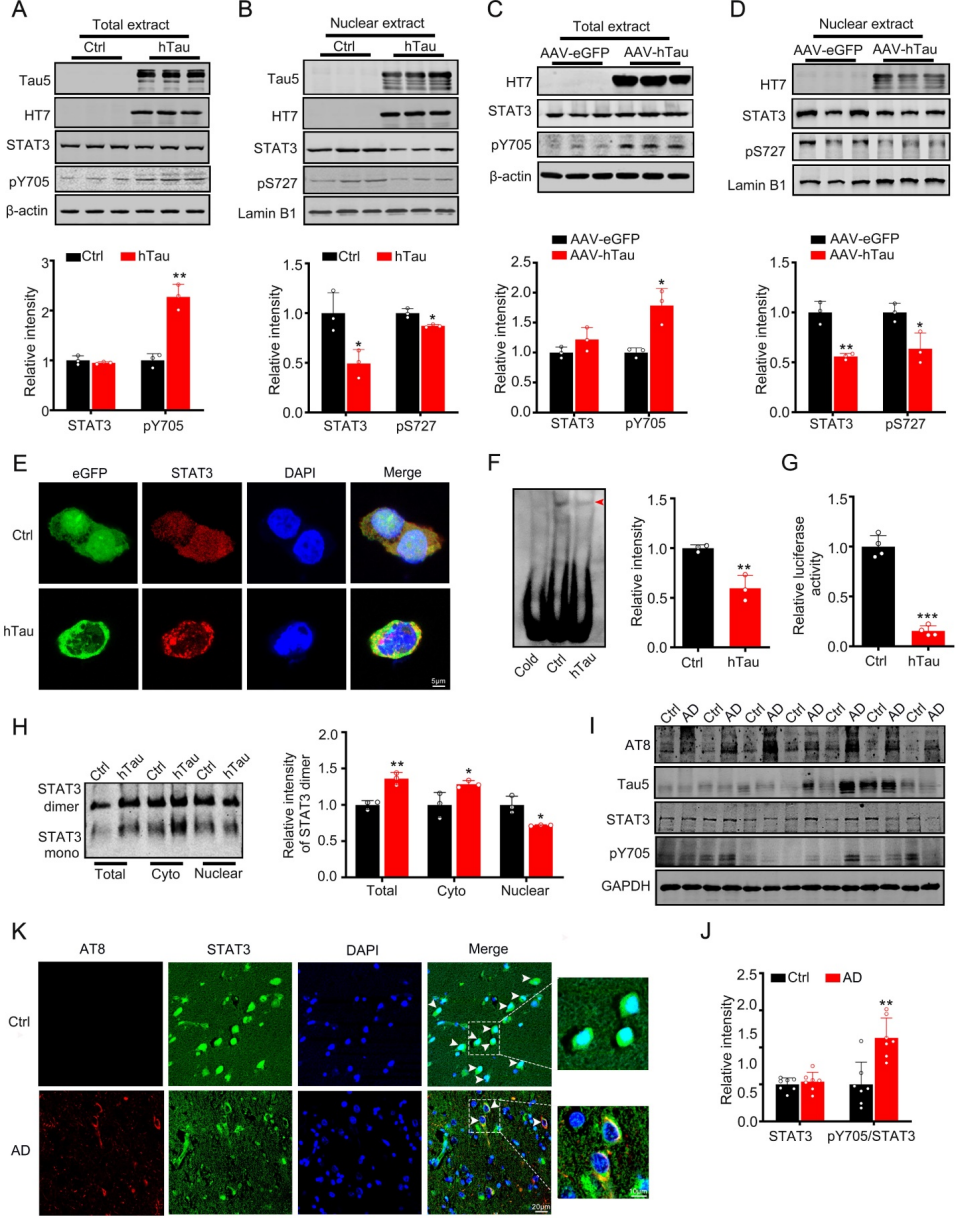
** Overexpression of hTau inactivates STAT3 via inhibiting its nuclear translocation.** (A, B) Expression of hTau increased the phosphorylated STAT3 at Tyr705 (pY705) in whole-cell extracts and decreased total and phosphorylated STAT3 at Ser727 (pS727) in the nuclear fraction detected by Western blotting (n = 3). The empty vector was transfected as a control (Ctrl). (C, D) AAV-hTau-eGFP (AAV-hTau) or the empty vector AAV- eGFP was stereotaxically injected into hippocampal CA3 of 2-month-old C57 mice. After 1 m, the increased levels of pY-STAT3 in hippocampal total extracts and decreased total and phosphorylated STAT3 (pS727) in the nuclear fraction were detected in hTau group by Western blotting (n = 3, Student's t-test). (E) The representative immunofluorescent images of STAT3 in HEK293 cells. Scale bar, 5 µm. (F) Overexpression of hTau decreased STAT3-DNA-binding activity in HEK293 cells measured by electrophoretic mobility shift assay (EMSA). Red arrowhead indicates STAT3/DNA complex. (G) Overexpressing hTau decreased STAT3 transcriptional activity in HEK293 cells detected by luciferase assay (n = 4). (H) Overexpressing hTau significantly decreased STAT3 dimer in nuclear fraction measured by Western blotting. (I, J) AT8 (pS202/pT205), STAT3 and pTyr705/STAT3 (pY705) in the cortex extracts of AD patients were measured by Western blotting (n = 7, Student's t-test). (K) The representative images of STAT3 in the brain of AD patients. Scale bar, 20 µm. Data were presented as mean ± SD. *, *p* < 0.05, **, *p* < 0.001, ***, *p* < 0.0001 *vs* Ctrl or eGFP.

As STAT3 is involved in the regulation of cognition 18-21, we detected the activity of STAT3 while hTau accumulation. We found that overexpression of hTau increased the phosphorylated level at Tyr705 of STAT3, which responses for STAT3 nuclear translocation and binding the promoter of target genes DNA sequence and activating them transcription 8, 9, while total STAT3 level in the cell extract in HEK293 cells had no change (Figure 1A). However, in the nuclear fraction, STAT3 level decreased and phosphorylated level at Ser 727 decreased, which suggests the inhibition of STAT3 activity (Figure 1B). Immunofluorescence image also confirmed STAT3 level decrease in the nucleus while hTau overexpression (Figure 1E). To further prove tau accumulation inactivating STAT3, transcriptional factor luciferase test, EMSA and Western Blotting (dimerization) were used. By EMSA using an oligonucleotide probe containing STAT3 binding site, we confirmed that hTau overexpression decreased the activity of STAT3 (Figure 1F), the association of STAT3 with DNA was disrupted by using cold probe, suggested the specificity of the method. The decrease activity of STAT3 was also proved by TF luciferase assay (Figure 1G), and the decrease dimerization of STAT3 in the nucleus was measured by Western blotting (Figure 1H). These *in vitro* data indicated that the intracellular hTau accumulation inhibited STAT3 activity.

AAV-hTau virus were injected into the bilateral hippocampus of 2-m-old C57 male mice to establish tau accumulated animal model, after one month, tau overexpression was proved by Western blotting (Figure 1C) and thioflavin S staining (Supplementary Figure S1). We also found that tau accumulation increased the pY705 STAT3 level in the total extract, and decreased total and phosphorylated level at Ser727 (pS727) of STAT3 in the nuclear fraction, while total STAT3 level in the total extract had no change (Figure 1C-D). By immunofluorescence, the nuclear STAT3 level was decreased in the neurons of brain slice of AD patients, where phosphorylated tau at pS202 and pT205 (AT8) was shown high immunoreaction (Figure 1K), with the ratio of pY705 STAT3/total STAT3 increased while the total STAT3 in the cortex of AD patients had no significant change (Figure 1I-J). These data suggested that hTau accumulation inhibited STAT3 translocation in the nucleus and inactivated STAT3 *in vivo* and *in vitro*.

### Intracellular hTau accumulation increases the interaction of STAT1 and STAT3 in the cytoplasm via STAT1 acetylation

**Figure 2:**
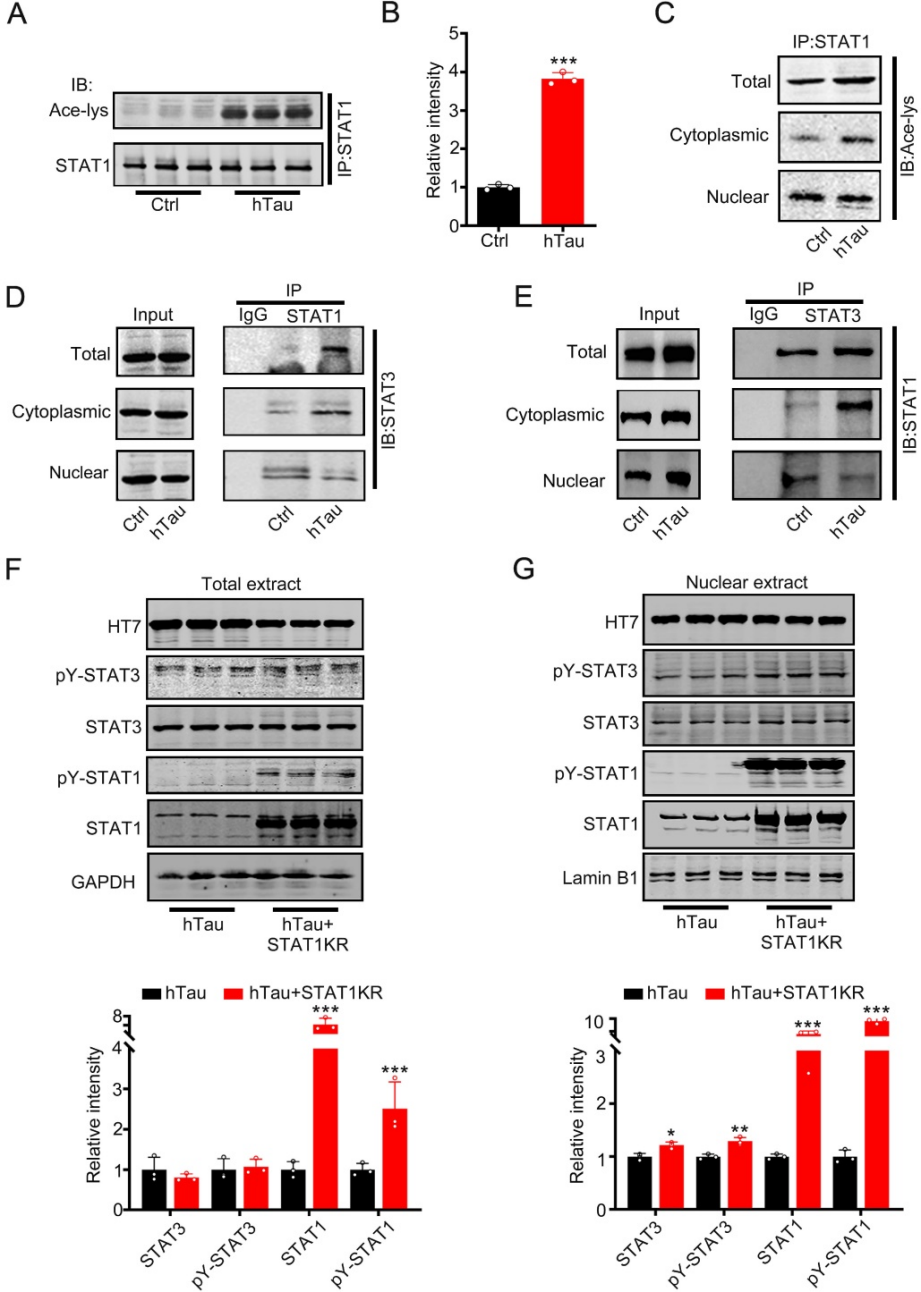
** Overexpression of hTau increases acetylated level of STAT1 by tau acetyltransferase activity.** (A-C) Overexpression of hTau increased the acetylation of STAT1 level in whole cell extracts and the cytoplasm, while decreased acetylated STAT1 in the nuclear extracts. Proteins were first immunoprecipitated with STAT1, and then detected with anti-ace-lysine antibody in whole cell, cytoplasmic and nuclear extracts by Western blotting. (D, E) Overexpression of hTau increased the interaction of STAT1 and STAT3 in the cytoplasmic fraction in HEK293 cells measured by CO-IP. (F, G) STAT1KR (K410/K413R-STAT1) restored hTau induced-decreased STAT3 in the nuclear fraction (B) and had no effects in STAT3 levels in total extracts measured by Western blotting (A) and quantitative analysis (n = 3). Data were presented as mean ± SD. *, *p* < 0.05, **, *p* < 0.001, ***, *p* < 0.0001 *vs* Ctrl or hTau.

Increased phosphorylated STAT3 at Tyr705 would induce STAT3 translocate from cytoplasm into the nucleus 8, 9, contradicted with the data that tau accumulation decreased STAT3 location in the nucleus. Krämer et al have reported that acetylated STAT1 is able to interact with NF-κB p65; as a consequence, p65 nuclear localization, DNA binding, and expression of anti-apoptotic NF-κB target genes all decreased 22. To probe for the cause, we hypothesize that hTau accumulation acetylated STAT1 to retain STAT3 in the cytoplasm by increasing the binding of STAT1 with STAT3 in the cytoplasm. Firstly, we detected STAT1 acetylation level while tau accumulation. Using immunoprecipitation, accumulating tau induced the acetylated level of STAT1 in the total extract and the cytoplasmic fraction increase significantly (Figure 2A-C). The binding of STAT1 and STAT3 in the total extract and cytoplasmic fraction also both increased, whereas the binding level decreased in the nuclear fraction while tau overexpression (Figure 2D-E). These data suggested that increased acetylated STAT1, which induced by hTau accumulation, increased its binding with STAT3 and detained STAT3 in the cytoplasmic fraction. As a consequence, STAT3 level decreased in the nuclear fraction. Our previous study has found that HDAC2, 4 and 5 levels had no change, and HAT activity decreased in hTau mice 23. Here, we also found that an intrinsic histone acetyltransferase p300 and H3K9 specific acetyltransferase PCAF level had no change while hTau overexpression (Supplementary Figure S2). Mammalian tau proteins possess intrinsic enzymatic activity capable of catalyzing self-acetylation 24, which facilitates tau lysine acetylation. We have found that acetyl transferase domain deleted tau had no effects in the acetylated level of STAT1 25, and non-acetylated STAT1 mutant (K410/413R-STAT1, STAT1KR) plasmid was transfected into HEK293 cells, and we found that STAT1KR overexpression attenuated hTau-induced decreased STAT3 level in the nuclear fraction (Figure 2G), while the total STAT3 level had no significant change (Figure 2F). All the data suggest that tau acetylated STAT1 directly, and STAT3 retained in the cytoplasm was due to increased level of STAT1 acetylation.

### STAT3 overexpression ameliorates hTau-induced synapse and memory impairments

**Figure 3:**
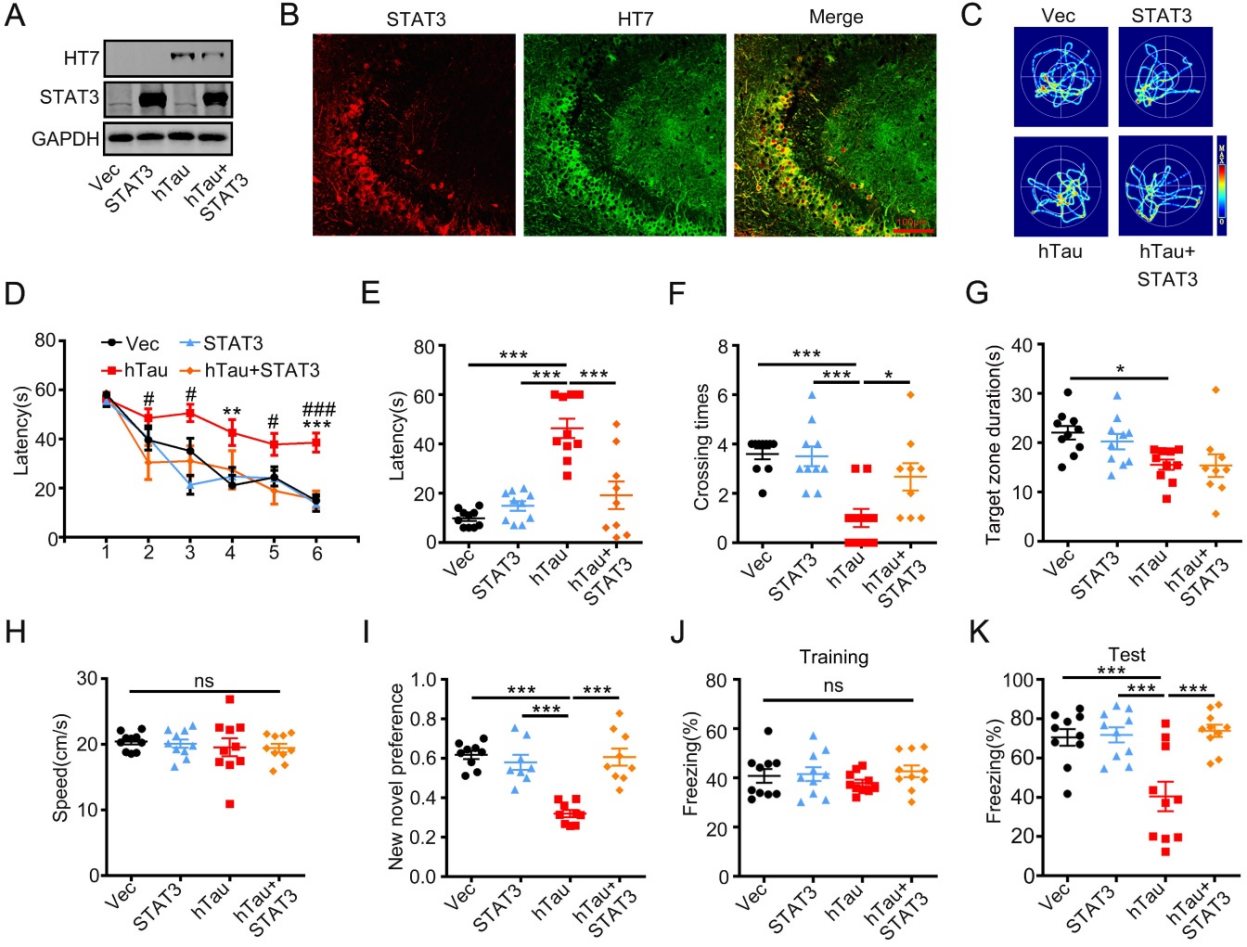
** Overexpression of STAT3 ameliorates hTau-induced memory deficits.** AAV-hTau-eGFP (1.5×10^13^ vg/mL) with AAV-STAT3 (1.2×10^13^ vg/mL) or AAV-vec were stereotaxically injected into the hippocampal CA3 of 2-m-old C57 mice. The learning and memory ability were detected 1 month later. (A, B) Upregulation of STAT3 was confirmed by Western blotting and immunofluorescent staining. (C) The representative swimming paths of each group during the test phase. (D) Upregulation of STAT3 ameliorated hTau-induced spatial learning deficits shown by the decreased escape latency during 6 consecutive days training in Morris water maze (MWM) test (N_Vec/STAT3/ hTau_ = 10, N_hTau+STAT3_ = 9). **, *p* < 0.01, ***, *p* < 0.001, hTau *vs* Vec; #, *p* < 0.05, ###, *p* < 0.001 hTau *vs* hTau+STAT3. (E-H) Upregulation of STAT3 ameliorated hTau-induced spatial memory deficits shown by the decreased latency to reach the platform quadrant (E), and crossing time in the platform site (F) measured at day 7 by removed the platform in MWM test; STAT3 overexpression had no effect in the time spent in the target quadrant (G) and the swimming speed had no change (H) (N_Vec/STAT3/ hTau_ = 10, N_hTau+STAT3_ = 9). (I) Upregulation of STAT3 ameliorated hTau-induced cognition impairment shown by increased time spending in exploring the new novel object measured at 24 h during New Novel object recognition test (N_Vec/STAT3/ hTau_ = 9, N_hTau+STAT3_ = 8). (J, K) Upregulation of STAT3 ameliorated hTau-induced contextual memory deficits measured at 24 h during contextual fear conditioning test (n = 10 each group). After the mice received training by subjecting to 3 min unsignaled foot-shocks, the percent of freezing time was represented (J). At 24 h after the training, the animals were put back into the conditioning chamber for 3 min (no electrical stimulation), the percent of total freezing time which represented memory ability was represented (K). Data were presented as mean ± SEM. *, *p* < 0.05, ***, *p* < 0.001.

To investigate whether overexpression of STAT3 ameliorates hTau-induced learning and memory deficits, AAV-STAT3 combined with AAV-hTau bilaterally injected into the hippocampus of 2-m-old C57 mice, and cognition was detected after 1 month. Western blotting and immunofluorescence showed the high transfected effects of AAV virus (Figure 3A-B). Using MWM test, hTau overexpression impaired learning abilities shown as the escape latency increased in 4^th^ and 6^th^ compared to those of the control, however, overexpressing STAT3 attenuated the learning deficits induced by hTau accumulation at 2^nd^, 3^rd^, 5^th^ and 6^th^ (Figure 3D). We also found that overexpressing STAT3 improved the hTau-induced memory impairments shown as less average latency to reach the previous target quadrant, more times across the target quadrant than the hTau mice (Figure 3C, E-F). To compare the time stayed in the platform quadrant, overexpressing STAT3 had no significant difference than the hTau group (Figure 3G). No significant difference in swimming speed was also seen among the four groups (Figure 3H), which excluded motor deficits. By new novel object test, STAT3 also attenuated hTau-induced long term memory deficits showed by more time to explore the new object (Figure 3I). By contextual fear conditioning test, we also observed that STAT3 overexpression rescued long term memory defects shown by an increased freezing time during memory test (Figure 3J-K). These data suggested that overexpressing STAT3 ameliorated hTau-induced cognitive deficits.

**Figure 4:**
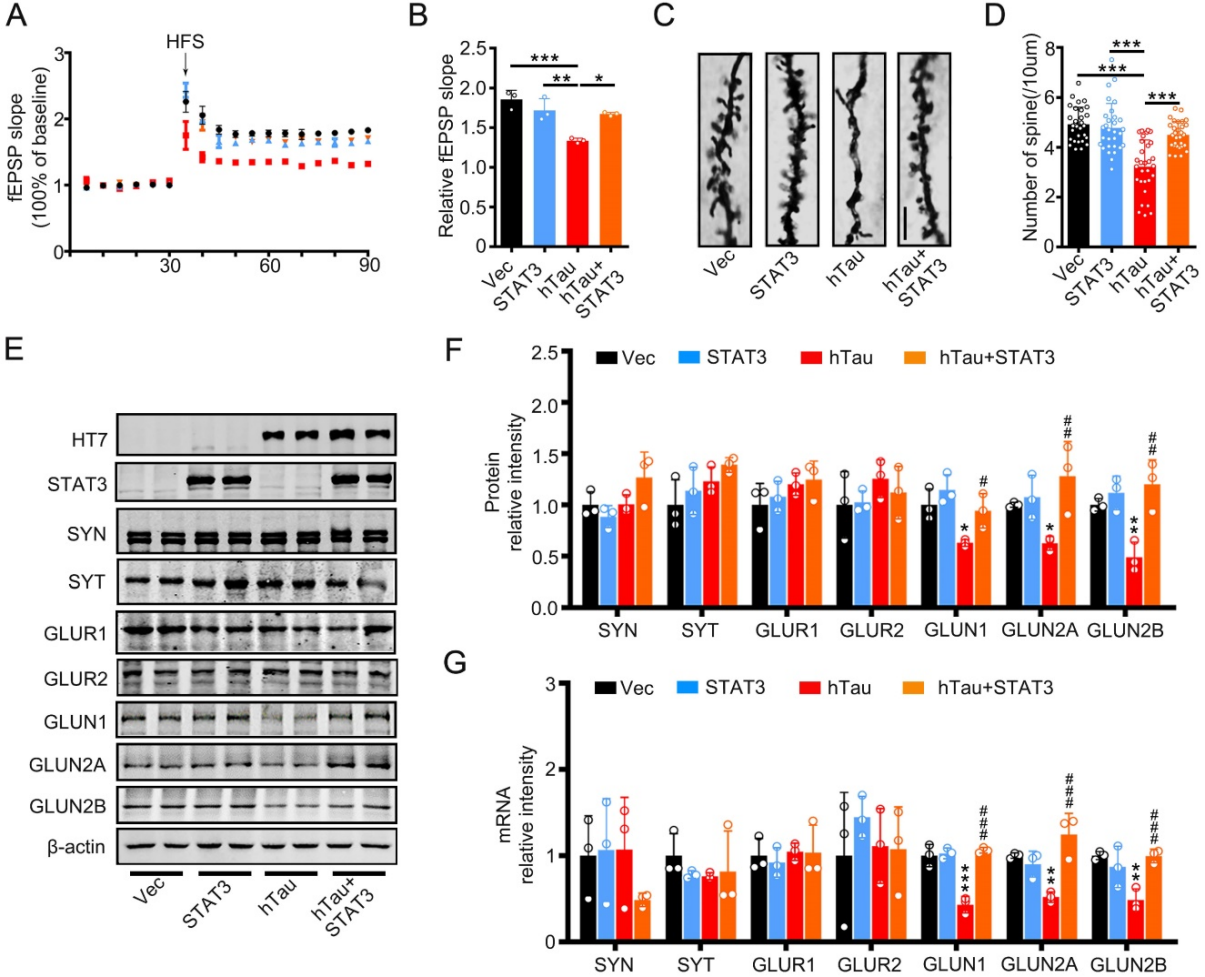
** Overexpression of STAT3 ameliorates hTau-induced synaptic impairments and decreased NMDARs level *in vivo*.** (A, B) Overexpression of STAT3 restored slopes of field excitatory postsynaptic potential (fEPSP) recorded in the hippocampal CA3 and quantitative analysis (B). (n = 5 slices from 3 mice for each group). * *p* < 0.05, **, *p* < 0.01, ***, *p* < 0.001. (C, D) Overexpression of STAT3 restored the density of dendritic spine detected by Golgi staining. The representive image (C) and quantitative analysis (D). Scale bar, 5 µm. Data were presented as mean ± SD. ***, *p* < 0.001. (E-G) Overexpression of STAT3 ameliorated the protein (E, F) and mRNA levels (G) of NMDARs, which detected by Western blotting and RT-PCR. Data were presented as mean ± SD*, *p* < 0.05, **, *p* < 0.01, ***, *p* < 0.001 *vs* Ctrl; #, *p* < 0.05, ##, *p* < 0.01, ###, *p* < 0.001 *vs* hTau.

To investigate the underlying mechanisms of the improvement effects of STAT3 in cognition, we detected the fEPSP slope, and found that the decreased fEPSP slope induced by hTau overexpression attenuated by overexpressing STAT3 (Figure 4A-B). hTau overexpression decreased the density of spine significantly compared with the control, whereas STAT3 ameliorated the reduction of hTau-induced spine density by Golgi staining (Figure 4C-D). Our previous study reported that hTau overexpression decreased NMDARs mRNA and protein levels 20. Here, in order to explore the molecular mechanisms of STAT3 attenuating hTau-induced cognitive deficits, we detected NMDARs level while overexpressing STAT3. We found that hTau decreased GluN1, GluN2A and GluN2B protein and mRNA levels, while overexpressing STAT3 ameliorated the reduction of hTau-induced NMDARs protein and mRNA levels (Figure 4E-G).

We also explore the effects of STAT3 overexpression in tau phosphorylation and aggregation, we found that the decrease human tau phosphorylated level at Ser396, Ser404, Thr231 and Ser205 were shown in soluble and insoluble fractions by overexpressing STAT3. These data suggest that overexpressing STAT3 could attenuate hTau-toxicities by reducing tau hyperphosphorylation and the pathological aggregation (Supplementary Figure S3).

### Inhibition of STAT1 acetylation rescues hTau-induced synapse and memory impairments

**Figure 5:**
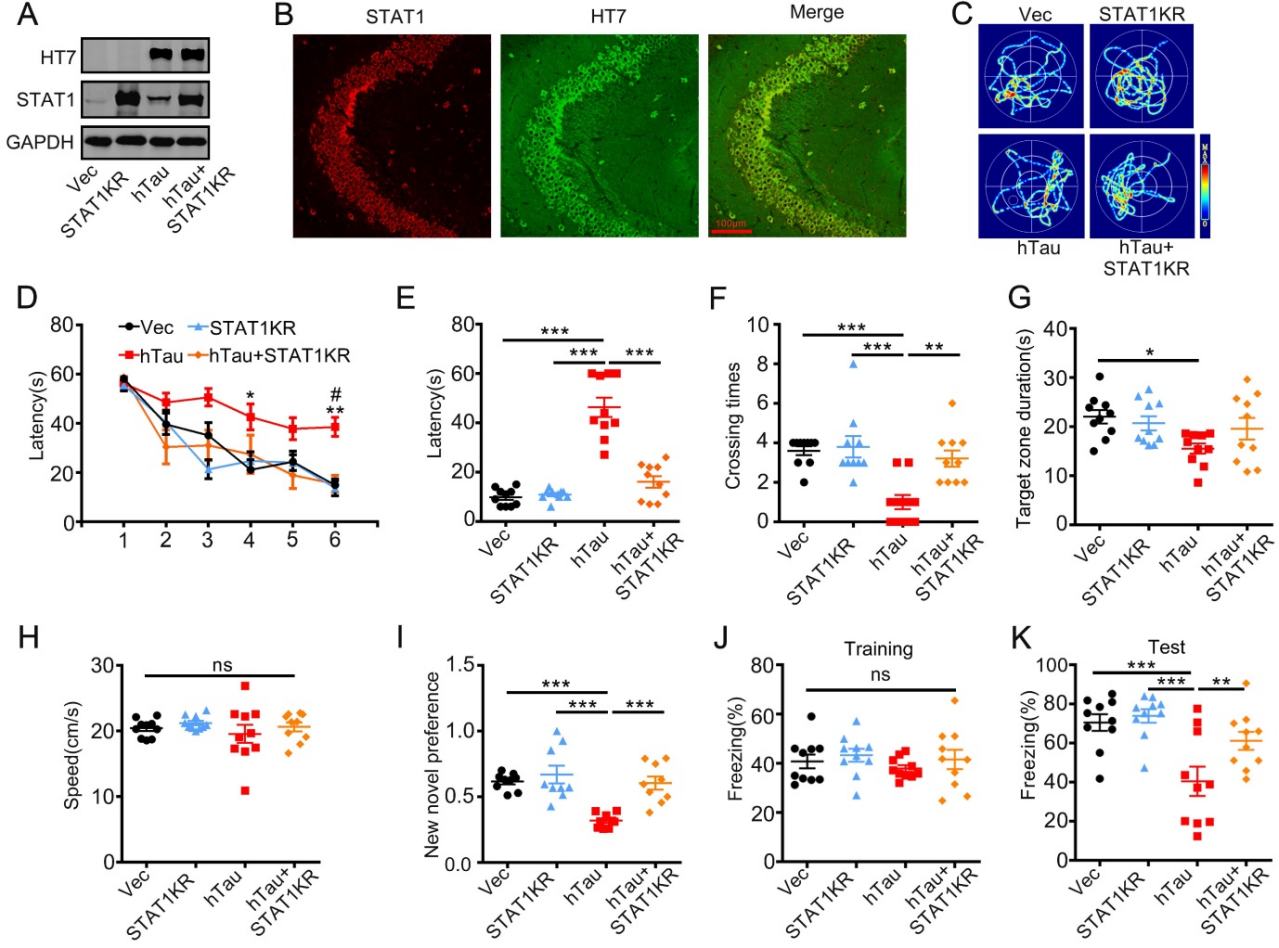
** Overexpression of STAT1KR ameliorates hTau-induced memory deficits.** AAV-hTau-eGFP (1.5×10^13^ vg/mL) with AAV- K410/K413R-STAT1 (STAT1KR), (1.0×10^13^ vg/mL) or not were stereotaxically injected into the hippocampal CA3 of 2-mon-old C57 mice. The learning and memory ability were detected 1 month later. (A, B) Upregulation of STAT1KR was confirmed by Western blotting and immunohistochemical staining. (C) The representative swimming paths of each group during the test phase. (D) Upregulation of STAT1KR ameliorated hTau-induced spatial learning deficits shown by the decreased escape latency during 6 consecutive days training in Morris water maze (MWM) test (n = 10 each group). *, *p* < 0.05, **, *p* < 0.01, hTau *vs* Vec; #, *p* < 0.05 hTau *vs* hTau+STAT1KR. (E-H) Upregulation of STAT1KR ameliorated hTau-induced spatial memory deficits shown by the decreased latency to reach the platform quadrant (E), and the increased crossing time in the platform site (F) measured at day 7 by removed the platform in MWM test; while the time spent in the target quadrant had no change, and no motor dysfunction was seen (H) (n = 10 each group). (I) Upregulation of STAT1KR ameliorated hTau-induced cognition impairment shown by increased time spending in exploring the new novel object measured at 24 h during New Novel object recognition test (n = 9 each group). (J, K) Upregulation of STAT1KR ameliorated hTau-induced contextual memory deficits measured at 24 h during contextual fear conditioning test (n = 10 each group). After the mice received training by subjecting to 3 min unsignaled foot-shocks, the percent of freezing time was represented (J). At 24 h after the training, the animals were put back into the conditioning chamber for 3 min (no electrical stimulation), the percent of total freezing time which represented memory ability was represented (K). Data were presented as mean ± SEM. *, *p* < 0.05, **, *p* < 0.01, ***, *p* < 0.001.

As acetylated STAT1 inhibited STAT3 nuclear translocation, and thus inactivated STAT3, a non-acetylated STAT1 virus AAV-KR-STAT1 (STAT1K410/413R, STAT1KR) were injected into the hippocampus of 2-m-old C57 mice with AAV-hTau, the animal's cognition was detected after one month. Western blotting and immunofluorescence showed the high transfected effects of AAV virus (Figure 5A, B). By MWM test, overexpressing STAT1KR improved the learning and memory abilities impairments induced by hTau shown as the escape latency was decreased in 6th day during the training stage (Figure 5D), and less escape latency to find the site where the platform put previous, more times across the target quadrant (Figure 5C, E-G). There was no significant difference in the swimming speed which excluded the motor deficits (Figure 5H). STAT1KR also attenuated hTau-induced long term memory showed by spent more time to explore the new object, and improved long-term memory shown by an increased freezing time during memory test in hTau combined with STAT1KR overexpressing mice, which detected by new novel object test or contextual fear conditioning test, respectively (Figure 5I-K).

**Figure 6:**
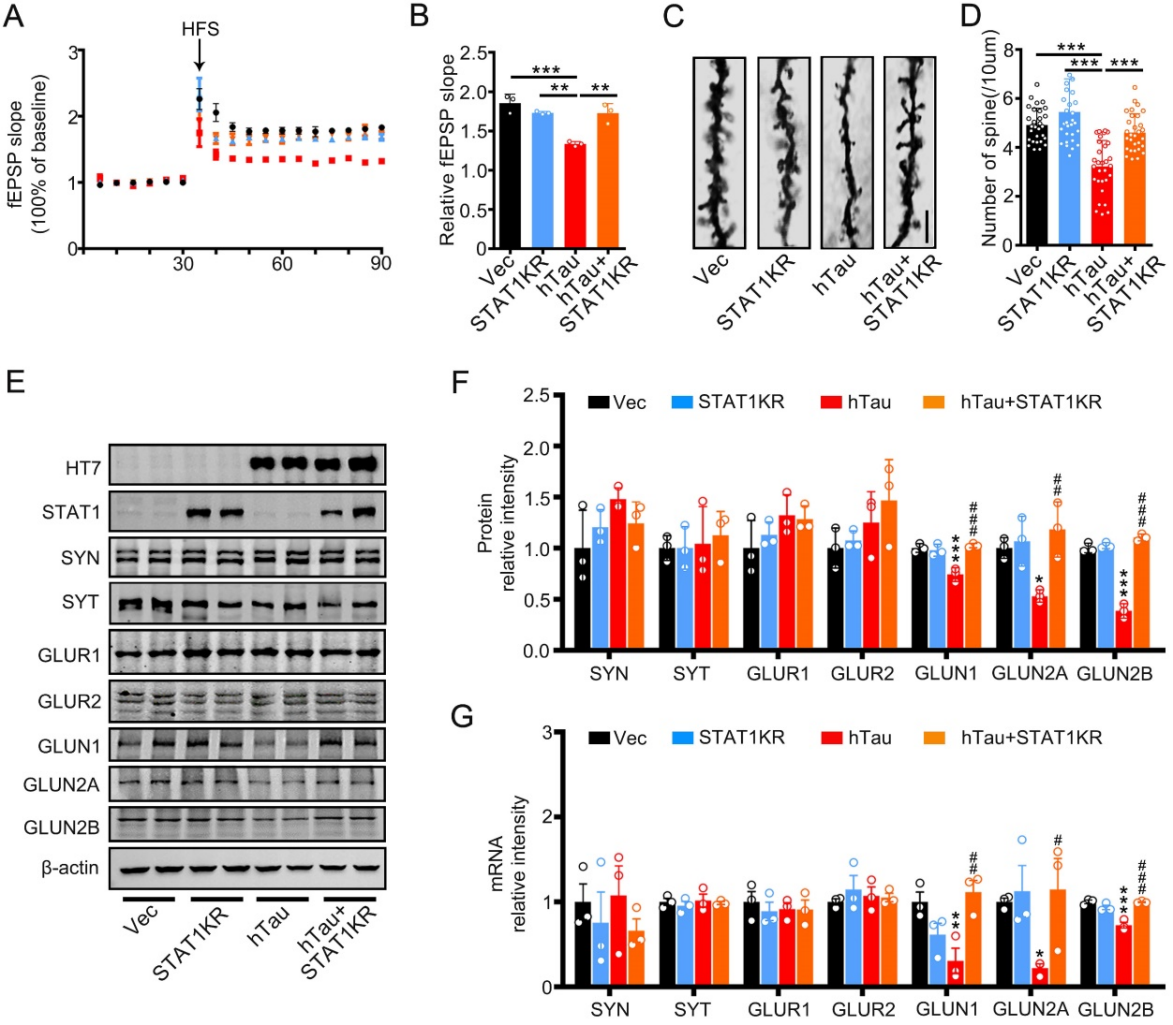
** Overexpression of Non-acetylated STAT1 ameliorates hTau-induced synaptic impairments and decreased NMDARs level *in vivo*.** (A, B) Overexpression of STAT1KR restored slopes of field excitatory postsynaptic potential (fEPSP) recorded in the hippocampal CA3 and quantitative analysis. (n = 5 slices from 3 mice for each group). (C, D) Overexpression of STAT1KR restored the density of dendritic spine detected by Golgi staining. The representive image (C) and quantitative analysis (D) Scale bar, 5 µm (n = 30 neurons from 3 mice for each group). Data were presented as mean ± SD. **, *p* < 0.01; ***, *p* < 0.001. (E-G) STAT1KR (AAV-K410/413R-STAT1) ameliorated the protein (E, F) and mRNA levels (G) of NMDARs, which detected by Western blotting and RT-PCR. Data were presented as mean ± SD. *, *p* < 0.05, **, *p* < 0.01, ***, *p* < 0.001 *vs* Ctrl; #, *p* < 0.05, ##, *p* < 0.01, ###, *p* < 0.001 *vs* hTau.

To explore the molecular mechanisms underlying STAT1KR improvement of cognitive deficits induced by hTau, we found that accumulating hTau decreased the spine density and fEPSP slope compare with the control, however, STAT1KR attenuated the reduction (Figure 6A-D). STAT1KR also ameliorated the reduction of protein and mRNA levels of NMDARs induced by hTau, which measured by Western blotting and RT-PCR (Figure 6E-G).

### Non-acetylated STAT1 induced STAT1 or STAT3 activity increase while Tau overexpression

**Figure 7:**
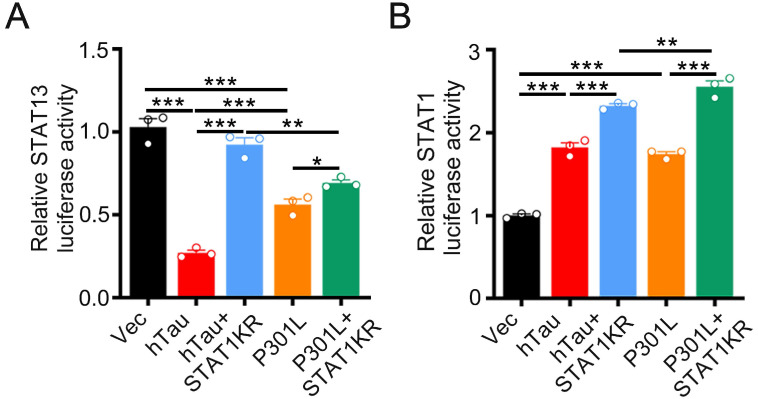
** STAT1KR activated STAT1 or STAT3 to different level while hTau or P301LhTau overexpression.** (A) STAT1KR increased the transcriptional activity of STAT3 higher in hTau than P301LhTau overexpression cells measured by luciferase activity assay (n = 3). (B) STAT1KR increased the transcriptional activity of STAT1 higher in P301LhTau than hTau overexpression cells measured by luciferase activity assay (n = 3). Data were presented as mean ± SD. *, *p* < 0.05, **, *p* < 0.001, ***, *p* < 0.0001.

By luciferase assay, overexpression of hTau or P301L-hTau decreased the transcription activity of STAT3, while overexpression STAT1KR (K410/413R-STAT1) attenuated the P301L-hTau or hTau induced-decreased STAT3 activity, and the activity of STAT3 increased higher in hTau than P301L-hTau overexpressed HEK293 cells (Figure 7A). We also found that overexpressing STAT1KR with P301L-hTau not hTau markedly increased higher transcription activity of STAT1 (Figure 7B).

## Discussion

It's well recognized that intracellular accumulation of hTau plays a critical role in the cognitive deficits of tauopathies, such as Alzheimer's disease, Parkinson disease and amyotrophic lateral sclerosis, and so on 26-28. Abnormal tau accumulation in the brain has a positively correlation with neurodegeneration and memory deterioration 29, 30. In AD patients, the total tau level in cerebrospinal fluids is inversely correlated with memory score 31, 32. Overexpression of hTau triggers memory impairment of the mice 33, 34, while inhibition of tau expression or immunotherapy against pathological tau ameliorates neuronal loss and memory deficits in hTau transgenic mice 35, 36. Knockout or reducing endogenous tau attenuates neuronal dysfunction and prevents behavioral deficits in transgenic mice expressing human amyloid precursor protein (APP) without altering Aβ level 37, 38. The global intensity of tau-PET, but not β-amyloid-PET signal predicted the rate of subsequent atrophy, independent of baseline cortical thickness 39. We also found that abnormal accumulated tau led to mitochondria dysfunction via inducing mitochondrial dynamics disorders and mitophagy deficits 40, 41. In the primary culture neurons, accumulated tau activated calcineurin to dephosphorylate CREB and calcium/calmodulin-dependent protein kinase IV, and thus caused intracellular calcium signaling disorders 33. Tau accumulation inhibits IST1 factor associated with ESCRT-III (IST1) expression to disrupt IST1-regulated ESCRT-III complex formation and impedes fusion of autophagosome with lysosome leading to autophagy deficits *in vitro* and *in vivo* 42. These studies strongly suggest that tau plays an important role in learning and memory disorders, and partially disclose the mechanisms underlying the toxic effects of tau. However, the molecular mechanisms underlying hTau-induced synapse impairment is not fully understood.

Recently, we have found that tau accumulation upregulated JAK2/STAT1 signaling to induce STAT1 translocation into nucleus and activate STAT1 to bind with the special GAS elements in the promoter of GluN1, GluN2A, or GluN2B, and thus repress the expression of NMDARs, which reveals a novel mechanism underlying hTau-induced synapse impairment and memory deficits 21. It's known that both STAT1 and STAT3 are highly expressed in the brain, and reported to involve in the ischemic stroke and cognitive regulation 16-19, 43-44. Therefore, study the role of STAT3 in the synaptic and cognitive deficits is an attractive idea. By overexpressing human P301L mutant tau, the human tau with the most common FTDP-17 mutation 45, we found that P301L mutant tau overexpression inhibited STAT3 translocation into the nucleus and inactivated STAT3 by increasing the interaction of acetylated STAT1 with STAT3 in the cytoplasm 25. Here, by overexpressing human full length tau to mimic intraneuronal tau accumulation as seen in the sporadic AD cases, we investigated whether STAT3 is also involved in hTau-induced cognitive deficits.

Using multiple measures including EMSA, dimerization, phosphorylation (pS727 STAT3 level) and luciferase activity assay, we provide strong evidence showing that STAT3 activity was inhibited by hTau accumulation, which contradicted with the increased phospho-STAT3 (Tyr705) in the present study. It's known that increased phospho-STAT3 (Tyr705) induces STAT3 nuclear translocation and increase DNA binding 8, 9. However, the level of STAT3 and phosphorylated STAT3 at (Ser727) in the nuclear fraction both decreased while hTau overexpression. Acetylated STAT1 at Lys 410 and Lys 413 is able to interact with NF-KB p65 in the cytoplasm, which led to p65 nuclear localization, DNA binding, and the decrease expressions of NF-KB target genes 22. Here, we found that hTau accumulation induced STAT1 acetylation and enhanced the interaction of STAT1 and STAT3 in the cytoplasm. By overexpressing KR-STAT1 mutant plasmid, we further confirmed that, accumulating hTau acetylated STAT1, and then, acetylated STAT1 was detained in the cytoplasm to binding with STAT3 to inhibit STAT3 translocate into nucleus.

In our previous papers, we found that STAT1 and STAT3 both regulate NMDAR expression through directly binding the GAS elements (TTC(N2-4) GAA for STAT1, and TTC(N3) GAA for STAT3) in the promoter of GluN1, GluN2A or GluN2B. However, like twins of Gemini of Greek mythology, we firstly found that STAT1 negative but STAT3 positive regulate NMDARs transcription 21, 25. Here, we also found that overexpressing STAT3 attenuated hTau-induced synaptic and memory impairments by increasing NMDAR expression. Combined with our published data, we conclude that inhibition of NMDAR expression by activation of STAT1 and inactivation of STAT3 may be a general mechanism of the synaptic and cognitive deficits in tauopathies.

We have found that non-acetylated STAT1 (STAT1KR) overexpression had no effects in the synaptic and memory impairments induced by P301L mutant human tau 25. In the present study, we surprised to find that STAT1KR attenuated synaptic and cognitive defects induced by tau accumulation. By luciferase test, we found that, compared with P301L-hTau combined with STAT1KR overexpression, hTau with STAT1KR overexpression increased higher transcriptional activity of STAT3 while smaller transcriptional activity of STAT1, which consistent with the data that STAT1KR ameliorated the hTau-induced decreased NMDAR transcription and expression. The positive effect of STAT3 was cancelled by the negative effect of STAT1 in the regulation of NMDAR transcription while overexpressing P301L-hTau and STAT1KR.

STAT3 is the main transcriptional enhancer of several autophagy-related genes in the nucleus 46, which executes its pro-autophagic function by upregulating hypoxia inducible factor 1 (HIF1A) and BNIP3 47, 48. Several microRNAs (such as MIR17HG, MIR21 and Mir30c) that target autophagy-related genes, are also regulated by nuclear STAT3 46, 49-51. Therefore, the reduction of soluble and insoluble tau observed in the current study may be due to the STAT3-stimulated autophagic degradation, which may deserve further investigation.

In conclusion, we find here that intracellular accumulation of hTau acetylated STAT1 to binding with STAT3, and then inhibit STAT3 translocate from cytoplasm to nucleus. Overexpressing STAT1KR or activation of STAT3 efficiently rescues the hTau-induced synaptic dysfunction and memory impairments in mice.

## Methods

### Antibodies and reagents

The antibodies used in the present study were listed in the Supplementary Table S1. The plasmid pEGFP-Tau-2N4R (Tau40), encoding human Tau, was a generous gift of Dr. Fei Liu (Jiangsu Key Laboratory of Neuroregeneration, Nantong, China). The wild type STAT1 and STAT3 (WT-STAT1, WT-STAT3) plasmids were gifts of Dr. Xiao-Yuan Li (Institute of Biomedical Sciences, Academia Sinica, Taiwan). WT-STAT1 plasmid was mutated to unacetylated STAT1 plasmids (double lysine site (410, 413) mutated to arginine, K410/3R-STAT1) coded into GFP-N1 vector with RFP-tag by Shanghai baicheng biotechnology co., LTD.

### Animals

Male C57 mice were purchased from the animal center of Tongji Medical College, Huazhong University of Science and Technology. All mice were kept at 24 ± 2 ºC on daily 12 h light-dark cycles with ad libitum access to food and water. All animal experiments were performed according to the 'Policies on the Use of Animals and Humans in Neuroscience Research' revised and approved by the Society for Neuroscience in 1995, and the Guidelines for the Care and Use of Laboratory Animals of the Ministry of Science and Technology of the People's Republic of China, and the Institutional Animal Care and Use Committee at Tongji Medical College. The animal study was approved by the Academic Review Board of Tongji Medical College, Huazhong University of Science and Technology.

### Stereotaxic brain injection

Adeno-associated virus coded for human full-length hTau with the N-terminal fused with enhanced green fluorescent protein (eGFP) or the control AAV-eGFP, AAV-STAT3, AAV-K410/3R-STAT1 virus were purchased from OBiO Biologic Technology Co., Ltd. The titer of AAV- hTau was 1.5×10^13^ vg/mL, that of AAV-STAT3 virus was 1.2×10^13^ vg/mL, and the titer for AAV-STAT1KR (K410/3R-STAT1) virus was 1.0×10^13^ vg/mL. All AAV viruses were driven by CMV-promoter. For brain injections, 2 m-old C57 mice were positioned respectively in a stereotaxic instrument, the virus was bilaterally injected into the hippocampal CA3 region (AP ± 2.0, ML -1.5, DV -2.0) at a rate of 0.10 μL/min. The needle syringe was left in place for 10 min before being withdrawn. The injection did not significantly increase the death rate or change the normal activity of the mice compared with the non-injected controls.

### Behavioral tests

Four weeks after brain infusion of the viral vectors, the spatial learning and memory were assessed by Morris water maze (MWM) test 42. For spatial learning, mice were trained in water maze to find a hidden platform placed in one quadrant for 6 consecutive days, 3 trials per day with a 30 s interval from 14:00 to 20:00 pm. The place of the platform didn't move during 6 consecutive days. On each trial, the mice started from one of the other three quadrants facing the wall of the pool and ended when the animal climbed on the platform. If the mice did not locate the platform within 60 s, they were guided to the platform and stayed for 20 s. The spatial memory was tested 1 day after the last training. The swimming path and the time used to find the platform (latency) or pass through the previous platform quadrant were recorded each day by a video camera fixed to the ceiling, 1.5 m from the water surface. The camera was connected to a digital-tracking device attached to an IBM computer.

The contextual fear conditioning test was performed as the procedures established in our lab 21. Briefly, the mouse was kept in the cage for 3 min to adapt to the environment before experiments, and then the mice received training by subjecting to 3 min unsignaled foot-shocks (0.5 mA, 2 s duration, and 1 min apart). The long-term memory (LTM) was tested respectively in 24 h after the training by subjecting back into the conditioning chamber for 3 min and measuring the freezing time.

The mice were habituated to the arenas (50 cm × 50 cm container) for 5 min without objects 24 h prior to the test. The day after the mice reentered the arenas from the same starting point, they were granted 5 min to explore two different objects A and B. After each familiarization period the arena and objects were cleaned with 70% ethanol. After one day, object A was replaced with another object C, and the mice were granted 5 min to explore both objects. The behavior was recorded by a video camera positioned above the arena. The recognition index was calculated by TC/ (TA + TC) 52.

### Electrophysiological analysis

Mice brains were cut into horizontal sections of 300 μm thickness by a vibration microtome in cold artificial cerebrospinal fluid (aCSF) containing NaCl 126 mM, KCl 2.5 mM, NaHCO_3_ 26 mM, NaH_2_PO_4_ 1.25 mM, CaCl_2_ 2 mM, MgCl_2_ 2 mM, glucose 10 mM, equilibrated with 95% O_2_ and 5% CO_2_. Immediately after slicing, sections were transferred in an interface chamber continuously perfused with aCSF. After 30 min, individual slices were laid down over an 8×8 array of planar microelectrodes, each 50 × 50 mm in size, with an interpolar distance of 450 mm (MED P5455; Alpha MED Sciences, Kadoma, Japan) and kept submerged in aCSF (4 mL/min; 32 °C) with a nylon mesh glued to a platinum ring. Voltage signals were acquired using the MED64 System (Alpha MED Sciences). Field excitatory postsynaptic potentials (fEPSPs) were recorded from CA3 in hippocampus by stimulating mossy fibers. Stimulation intensity was adjusted to evoke fEPSP amplitudes that were 30% of maximal size. For induction of long-term potentiation (LTP), theta-burst stimulation (TBS) was used which consisted of 3 pulses at 100 Hz, repeated 3 times with a 200 ms interval. The magnitudes of LTP are expressed as the mean percentage of baseline fEPSP initial slope.

### Human brain tissue

Postmortem human brain paraffin sections were provided by Dr. K Ye of the Emory University School of Medicine, USA. The human brain tissues were approved by Dr. Chao Ma of Peking Union Medical College, and Chinese Academy of Medical Sciences Human Brain Bank, Beijing, China. AD was diagnosed according to the criteria of the Consortium to Establish a Registry for AD and the National Institute on Aging. All subjects signed informed consent. The patients' information was listed in Supplementary Table S2.

### Western blotting

The HEK293 cells and C57BL/6J mice brain tissues were lysed in RIPA buffer (Beyotime, Shanghai, China) on ice for 10 min to collect the total cellular proteins. The nuclear and cytoplasmic proteins were fractionated using the Nuclear Extraction Kit (Signosis) by following the manufacturer's instructions. The protein concentration was determined by using BCA Protein Assay Kit. After electrophoresis, the proteins were transferred onto the nitrocellulose membrane, following blocked using 5% BSA for 1 h at room temperature. The membranes were incubated with primary antibody (Supplementary Table S1) overnight, and the immunoreactive bands were visualized by using the Odyssey Infrared Imaging System (LI-COR Biosciences, Lincoln, NE, USA).

### Immunofluorescence

Paraffin tissue samples were continuously sliced in 4 μm, and baked at 72 °C for 30 min. After the xylene was dewaxed, gradient ethanol was used for rehydration. Heat-induced sodium citrate antigen retrieval was performed using pH 6.0 sodium citrate at 95 °C for 20 min. Then the sections were membrane ruptured using 0.5% Triton X-100 for 30 min at room temperature, followed by incubated in 5% BSA for 30 min at 37 °C. Human brain slices were blocked with Sudan black B at room temperature for 15 min. Sections were then incubated with primary antibodies at 4 °C overnight. The secondary antibodies conjugated to Alexa Fluor 488/546 (Thermo Fisher Scientific) were added to the slices for 1 h at 37 °C. Finally, DAPI was applied for nuclear staining for 10 min at room temperature. The images were observed with a laser confocal microscope (710; Zeiss, Germany).

### Electrophoresis mobility shift assay (EMSA)

The EMSA-STAT3 (Signosis, USA) was performed according to the protocol supplied by the manufacturer. The nuclear samples were incubated with a biotinized oligonucleotide probe containing a STAT3 binding site at 22 °C for 30 min. Then, the samples were separated on a non-denaturing polyacrylamide gel and transferred to Nylon Membrane. The transferred oligonucleotides were immobilized by UV cross-linking at 120,000 J for 1 min. For detection, streptavidin-HRP was added to the membrane, and the blots were developed by ECL. Competition experiment was performed using excess amounts of unlabeled cold probe containing STAT3 binding site.

### Reverse transcription and real-time quantitative PCR

Total RNA was extracted from mouse CA3 hippocampus samples using Trizol reagent (Life Technologies, Carlsbad, CA). The reverse transcription and quantitative PCR was performed according to manufacturer's instruction (TaKaRa, Dalian, China). The PCR system contains 0.5 μM forward and reverse primers, 2 μL SYBR Green PCR master mixes and 2 μL cDNA, and the standards for each gene were prepared using appropriate primers by a conventional PCR. RT-PCR was performed in a StepOnePlus Real-Time PCR Detection System (Life Technologies, NY, USA). The expression level of the interest gene was normalized by the housekeeping gene β-actin, which was not changed by the treatments. PCR primers employed in the present study were listed in the supplementary Table S3.

### Luciferase reporter assay

The HEK293 cells were transfected with hTau plasmid or its empty vector control in combination with pSTAT3-Luc reporter construct and pRL-TK for 48 h. Then the cells were washed with PBS and lysed in 1 × CCLR for 10 min. The supernatant of the lysate was used to measure luciferase reporter gene expression using the Dual-Luciferase Reporter Assay System (Promega, USA). The activity of TF (i.e. firefly luciferase) was normalized to transfection efficiency by using Renilla luciferase activity (pRL-TK).

To generate luciferase reporter plasmids of GluN1, GluN2A or GluN2B promoter, PCR fragments from the mouse genomic DNA were inserted into the BglII and NcoI sites of the pGL3 basic luciferase expression vector (Promega, Madison, WI) 21. HEK293 cells were transfected with luciferase reporter plasmids. The luciferase activity reflects the transcriptional activity of the target genes. To assay the luciferase activity, cell extracts were used for luciferase activity assay using the Dual Luciferase Reporter (DLR) assay system (Promega) and Lumat LB9507 luminometer (Berthold).

### Immunoprecipitation

The HEK293 cell was homogenized on ice in RIPA buffer (Beyotime, Shanghai, China) for 10 min at 4 °C and then centrifuged at 12,000 × g for 15 min. 200 μg total proteins were incubated with primary antibodies (Supplementary Table S1) at 4 °C overnight followed by addition of protein G agarose (Millipore, Darmstadt, Germany) for 4 h (at 4 °C on rotation). Finally, the sample was collected for western blotting.

### Golgi impregnation

For Golgi stain, the mice were anesthetized and then perfused transcardially with 0.9% NaCl, and the brain tissues were incubated in 0.4% OsO_4_ and 3.5% K_2_Cr_2_O_7_ at room temperature for two days. The sections were then incubated in 1% AgNO_3_(aq) for one month at room temperature in dark. Then the slide assemblies were dismantled in water and the sections were mounted on gel-coated slides (0.5% porcine gelatin), dehydrated in gradient alcohol, counterstained with hematoxylin. The images were taken using Olympus BX60 (Tokyo).

### Statistical analysis

All data were collected and analyzed in a blinded manner. Data were expressed as mean ± SD or mean ± SEM and analyzed using SPSS 12.0 statistical software (SPSS Inc. Chicago, IL, USA). Statistical analysis was performed using Student's t-test, one-way ANOVA or two-way repeated measures ANOVA followed by Bonferroni's post hoc test. The level of significance was set at p < 0.05.

## Supplementary Material

Supplementary figures and tables.

## References

[1] Kisseleva T, Bhattacharya S, Braunstein J, Schindler CW (2002). Signaling through the JAK/STAT pathway, recent advances and future challenges. Gene.

[2] Wegenka UM, Buschmann J, Lütticken C, Heinrich PC, Horn F (1993). Acute-phase response factor, a nuclear factor binding to acute-phase response elements, is rapidly activated by interleukin-6 at the posttranslational level. Mol Cell Biol.

[3] Sadowski HB, Shuai K, Darnell JE Jr, Gilman MZ (1993). A common nuclear signal transduction pathway activated by growth factor and cytokine receptors. Science.

[4] Liddle FJ, Alvarez JV, Poli V, Frank DA (2006). Tyrosine phosphorylation is required for functional activation of disulfide-containing constitutively active STAT mutants. Biochemistry.

[5] Darnell JE, Kerr IM, Stark GR (1994). Jak-STAT pathways and transcriptional activation in response to IFNs and other extracellular signaling proteins. Science.

[6] Ihle JN (1995). Cytokine receptor signalling. Nature.

[7] Levy DE, Lee CK (2002). What does Stat3 do?. J Clin Invest.

[8] Levy DE, Darnell J (2002). Stats: transcriptional control and biological impact. Nat Rev Mol Cell Biol.

[9] Yu H, Lee H, Herrmann A, Buettner R, Jove R (2014). Revisiting STAT3 signalling in cancer: new and unexpected biological functions. Nat Rev Cancer.

[10] Priego N, Zhu L, Monteiro C, Mulders M, Wasilewski D, Bindeman W (2018). STAT3 labels a subpopulation of reactive astrocytes required for brain metastasis. Nat Med.

[11] Damasceno LEA, Prado DS, Veras FP, Fonseca MM, Toller-Kawahisa JE, Rosa MH (2020). PKM2 promotes Th17 cell differentiation and autoimmune inflammation by fine-tuning STAT3 activation. J Exp Med.

[12] Zhang Y, Ma CA, Lawrence MG, Break TJ, O'Connell MP, Lyons JJ (2017). PD-L1 up-regulation restrains Th17 cell differentiation in STAT3 loss and STAT1 gain-of-function patients. J Exp Med.

[13] Hsu WL, Ma YL, Hsieh DY, Liu YC, Lee EH (2014). STAT1 Negatively Regulates Spatial Memory Formation and Mediates the Memory-Impairing Effect of Aβ. Neuropsychopharmacology.

[14] Tai DJ, Hsu WL, Liu YC, Ma YL, Lee EH (2011). Novel role and mechanism of protein inhibitor of activated STAT1 in spatial learning. EMBO J.

[15] Park SJ, Shin EJ, Min SS, An J, Li Z, Hee Chung Y (2013). Inactivation of JAK2/STAT3 signaling axis and downregulation of M1 mAChR cause cognitive impairment in klotho mutant mice, a genetic model of aging. Neuropsychopharmacology.

[16] Yamada M, Chiba T, Sasabe J, Terashita K, Aiso S, Matsuoka M (2008). Nasal Colivelin treatment ameliorates memory impairment related to Alzheimer's disease. Neuropsychopharmacology.

[17] Chiba T, Yamada M, Sasabe J, Terashita K, Shimoda M, Matsuoka M (2009). Amyloid-β causes memory impairment by disturbing the JAK2/STAT3 axis in hippocampal neurons. Mol Psychiatry.

[18] Nicolas CS, Peineau S, Amici M, Csaba Z, Fafouri A, Javalet C (2012). The Jak/STAT pathway is involved in synaptic plasticity. Neuron.

[19] Szczepanek K, Lesnefsky EJ, Larner AC (2012). Multi-tasking: nuclear transcription factors with novel roles in the mitochondria. Trends Cell Biol.

[20] Chiba T, Yamada M, Sasabe J, Terashita K, Shimoda M, Matsuoka M (2009). Amyloid-beta causes memory impairment by disturbing the JAK2/STAT3 axis in hippocampal neurons. Mol Psychiatry.

[21] Li XG, Hong XY, Wang YL, Zhang SJ, Zhang JF, Li XC (2019). Tau accumulation triggers STAT1-dependent memory deficits by suppressing NMDA receptor expression. EMBO Rep.

[22] Krämer OH, Baus D, Knauer SK, Stein S, Jäger E, Stauber RH (2006). Acetylation of Stat1 modulates NF-κB activity. Genes Dev.

[23] Chai GS, Feng Q, Wang ZH, Hu Y, Sun DS, Li XG (2017). Downregulating ANP32A rescues synapse and memory loss via chromatin remodeling in Alzheimer model. Mol Neurodegener.

[24] Cohen TJ, Friedmann D, Hwang AW, Marmorstein R, Lee VM (2013). The microtubule-associated tau protein has intrinsic acetyltransferase activity. Nat Struct Mol Biol.

[25] Hong XY, Wan HL, Li T, Zhang BG, Li XG, Wang X (2020). STAT3 ameliorates cognitive deficits by positively regulating the expression of NMDARs in a mouse model of FTDP-17. Signal Transduct Target Ther.

[26] Tolnay M, Probst A (2003). The neuropathological spectrum of neurodegenerative tauopathies. IUBMB Life.

[27] Lee MY, Trojanowski JQ (1999). Neurodegenerative tauopathies: human disease and transgenic mouse models. Neuron.

[28] Lee VM, Goedert M, Trojanowski JQ (2001). Neurodegenerative tauopathies. Annu Rev Neurosci.

[29] Thal DR, Holzer M, Rüb U, Waldmann G, Günzel S, Zedlick D (2000). Alzheimer-related τ-pathology in the perforant path target zone and in the hippocampal stratum oriens and radiatum correlates with onset and degree of dementia. Exp Neurol.

[30] Yin Y, Gao D, Wang Y, Wang ZH, Wang X, Ye J (2016). Tau accumulation induces synaptic impairment and memory deficit by calcineurin-mediated inactivation of nuclear CaMKIV/CREB signaling. Proc Natl Acad Sci U S A.

[31] Hu YY, He SS, Wang X, Duan QH, Grundke-Iqbal I, Iqbal K (2002). Levels of nonphosphorylated and phosphorylated tau in cerebrospinal fluid of Alzheimer's disease patients: an ultrasensitive bienzyme-substrate-recycle enzyme-linked immunosorbent assay. Am J Pathol.

[32] Lin YT, Cheng JT, Yao YC (2009). Increased Total TAU but not Amyloid-β 42; in Cerebrospinal Fluid Correlates with Short-Term Memory impairment in Alzheimer's Disease. J Alzheimers Dis.

[33] Götz J, Deters N, Doldissen A, Bokhari L, Ke Y, Wiesner A (2007). A decade of tau transgenic animal models and beyond. Brain Pathol.

[34] Kimura T, Yamashita S, Fukuda T, Park JM, Murayama M, Mizoroki T (2007). Hyperphosphorylated tau in parahippocampal cortex impairs place learning in aged mice expressing wild-type human tau. EMBO J.

[35] Boutajangout A, Quartermain D, Sigurdsson EM (2010). Immunotherapy targeting pathological tau prevents cognitive decline in a new tangle mouse model. J Neurosci.

[36] Roberson ED, Scearce-Levie K, Palop JJ, Yan F, Cheng IH, Wu T (2007). reducing endogenous tau ameliorates amyloid ß-induced deficits in an Alzheimer's disease mouse model. Science.

[37] Ittner LM, Ke YD, Delerue F, Bi M, Gladbach A, van Eersel J (2010). Dendritic function of tau mediates amyloid-β toxicity in Alzheimer's disease mouse models. Cell.

[38] Vossel KA, Zhang K, Brodbeck J, Daub AC, Sharma P, Finkbeiner S (2010). Tau reduction prevents Aβ-induced defects in axonal transport. Science.

[39] La Joie R, Visani AV, Baker SL, Brown JA, Bourakova V, Cha J (2020). Prospective longitudinal atrophy in Alzheimer's disease correlates with the intensity and topography of baseline tau-PET. Sci Transl Med.

[40] Hu Y, Li XC, Wang ZH, Luo Y, Zhang X, Liu XP (2016). Tau accumulation impairs mitophagy via increasing mitochondrial membrane potential and reducing mitochondrial Parkin. Oncotarget.

[41] Li XC, Hu Y, Wang ZH, Luo Y, Zhang Y, Liu XP (2016). Human wild-type full-length tau accumulation disrupts mitochondrial dynamics and the functions via increasing mitofusins. Sci Rep.

[42] Feng Q, Luo Y, Zhang XN, Yang XF, Hong XY, Sun DS (2020). MAPT/Tau accumulation represses autophagy flux by disrupting IST1-regulated ESCRT-III complex formation: a vicious cycle in Alzheimer neurodegeneration. Autophagy.

[43] Li Z, Song Y, He T, Wen R, Li Y, Chen T (2021). M2 microglial small extracellular vesicles reduce glial scar formation via the miR-124/STAT3 pathway after ischemic stroke in mice. Theranostics.

[44] Qin C, Liu Q, Hu ZW, Zhou LQ, Shang K, Bosco DB (2018). Microglial TLR4-dependent autophagy induces ischemic white matter damage via STAT1/6 pathway. Theranostics.

[45] Lewis J, McGowan E, Rockwood J, Melrose H, Nacharaju P, Van Slegtenhorst M (2000). Neurofibrillary tangles, amyotrophy and progressive motor disturbance in mice expressing mutant (P301L) tau protein. Nat Genet.

[46] You L, Wang Z, Li H, Shou J, Jing Z, Xie J (2015). The role of STAT3 in autophagy. Autophagy.

[47] Jung JE, Kim HS, Lee CS, Shin YJ, Kim YN, Kang GH (2008). STAT3 inhibits the degradation of HIF-1alpha by pVHL-mediated ubiquitination. Exp Mol Med.

[48] Pratt J, Annabi B (2014). Induction of autophagy biomarker BNIP3 requires a JAK2/STAT3 and MT1-MMP signaling interplay in Concanavalin-A-activated U87 glioblastoma cells. Cell Signal.

[49] Brock M, Trenkmann M, Gay RE, Michel BA, Gay S, Fischler M (2009). Interleukin-6 modulates the expression of the bone morphogenic protein receptor type II through a novel STAT3-microRNA cluster 17/92 pathway. Circ Res.

[50] Nguyen HT, Dalmasso G, Müller S, Carrière J, Seibold F, Darfeuille-Michaud A (2014). Crohn's disease-associated adherent invasive Escherichia coli modulate levels of microRNAs in intestinal epithelial cells to reduce autophagy. Gastroenterology.

[51] Zhou X, Ren Y, Liu A, Han L, Zhang K, Li S (2014). STAT3 inhibitor WP1066 attenuates miRNA-21 to suppress human oral squamous cell carcinoma growth *in vitro* and *in vivo*. Oncol Rep.

[52] Shentu YP, Huo Y, Feng XL, Gilbert J, Zhang Q, Liuyang ZY (2018). CIP2A Causes Tau/APP Phosphorylation, Synaptopathy, and Memory Deficits in Alzheimer's Disease. Cell Rep.

