# Supplementary material for: STAT3 ameliorates cognitive deficits via regulation of NMDAR expression in an Alzheimer's disease animal model
Source: Theranostics. 2021 Mar 13;11(11):5511–24. doi: 10.7150/thno.56541 (PMC8039956; doi:10.7150/thno.56541)
Supplement: Supplementary file 1 — Supplementary figures and tables. [file thnov11p5511s1.pdf]

Supplementary materials for

STAT3 ameliorates cognitive deficits via regulation of NMDAR expression in an Alzheimer's disease animal model

Hua-Li Wan[1]‡, Xiao-Yue Hong[1]‡, Zai-Hua Zhao[2]‡, Ting Li[1], Bing-Ge Zhang[1], Qian Liu[1], Qun Wang[1], Shi Zhao[3], Jian-Zhi Wang[1,4], Xue-Feng Shen[2]*, Gong-Ping Liu[1,4]*

Correspondence to: Xue-Feng Shen (xfshen@fmmu.edu.cn) or Gong-Ping Liu (liugp111@mail.hust.edu.cn)

This PDF File includes:

Materials and Methods

Figures S1 to S3

Tables S1 to S3

Materials and Methods

Thioflavin-S staining

The brain sections were incubated in 0.25% potassium permanganate solution for 20 min, and then incubated in bleaching solution (2% oxalic acid and 1% potassium metabisulfite in distilled water) for 2 min. The sections incubated in blocking solution (1% sodium hydroxide and 0.9% hydrogen peroxide in distilled water) for 20 min. After incubated for 5 s in 0.25% acidic acid, 0.0125% Thioflavin-S (Sigma) in 50% ethanol was used for 5 min [1]. Finally, the sections were washed with 50% ethanol. The images were observed with a laser confocal microscope (710; Zeiss, Germany).

Western blotting

The HEK293 cells and C57BL/6J mice brain tissues were lysed in RIPA buffer (Beyotime, Shanghai, China) on ice for 10 min to collect the total cellular proteins. The nuclear and cytoplasmic proteins were fractionated using the Nuclear Extraction Kit (Signosis) by following the manufacturer's instructions. The protein concentration was determined by using BCA Protein Assay Kit. After electrophoresis, the proteins were transferred onto the nitrocellulose membrane, following blocked using 5% BSA for 1 h at room temperature. The membranes were incubated with primary antibody (Supplementary Table S1) overnight, and the immunoreactive bands were visualized by using the Odyssey Infrared Imaging System (LI-COR Biosciences, Lincoln, NE, USA).

Preparation of insoluble tau

Mice CA3 hippocampus samples were homogenized in lysis buffer (10 mM Tris-HCl, 150 mM NaCl, 20 mM NaF, 1 mM Na_3VO_4, 2 mM EGTA, 0.5% Triton X-100, and 0.1% SDS) with Protease and phosphatase inhibitor cocktail on ice for 10 min. The homogenate was centrifuged at 13,000 g for 20 min. After centrifugation, the supernatant was soluble tau fraction. The pellet was homogenized in 1% SDS buffer which was designated as insoluble tau protein [1].

Reference

[1] Li XG, Hong XY, Wang YL, Zhang SJ, Zhang JF, Li XC, et al. Tau accumulation triggers STAT1-dependent memory deficits by suppressing NMDA receptor expression. EMBO Rep. 2019; 20: e47202.

Supplementary Figures

Figure S1

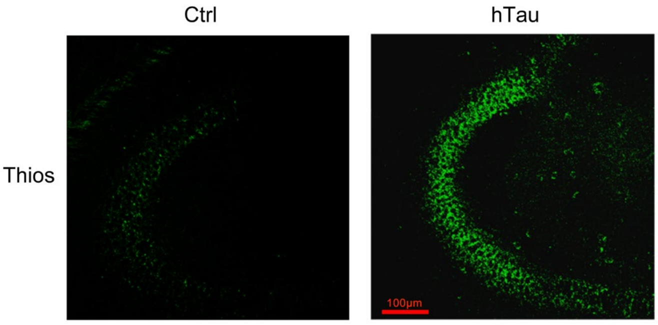

Figure S1 Overexpressing hTau induces tau aggregation.

AAV- hTau-eGFP or AAV-eGFP were stereotaxically injected into hipocampal CA3 of 2-month-old C57 mice. After 1 month, the representative images of Thioflavin S staining showed tau aggregation in the hippocampus of the virus-injected mice.

Figure S2

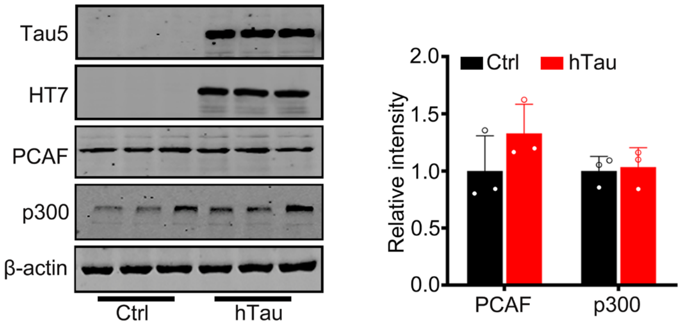

Figure S2 Overexpressing hTau has no effect on the protein level of acetylase PCAF or p300.

PCAF and p300 had no change while overexpressing hTau in HEK293 cells detected by Western blotting.

Figure S3

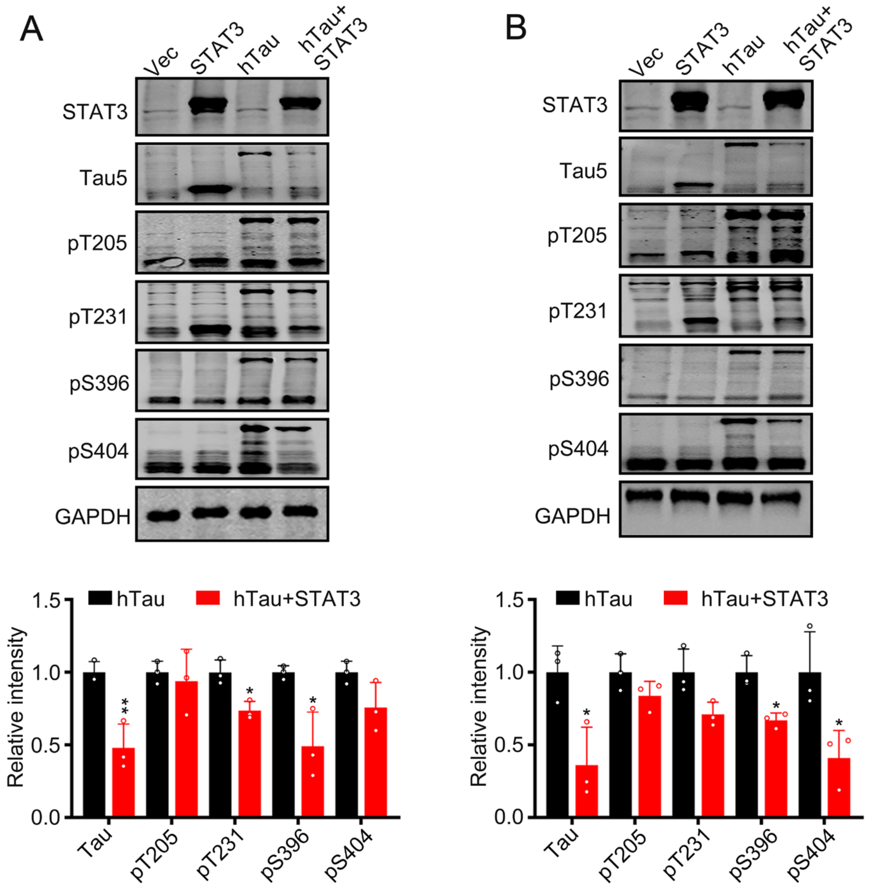

Figure S3 STAT3 decreases soluble or insoluble tau level.

AAV-hTau-eGFP with AAV-STAT3 or not were stereotaxically injected into the hippocampal CA3 of 2-mon-old C57 mice. After one month, levels of soluble (A) and insoluble phosphorylated tau (B) were detected by Western blotting.

Data were presented as mean ± SD. *, $p < 0.05$, **, $p < 0.01$ *vs* hTau.

Table S1 The antibodies used in the study

Antibody	Specificity	Type	Dilution	Source
STAT3	Total STAT3	Mono-	1:100 for IF<br>1:1000 for WB	Cell Signaling (Boston, MA)
STAT3	Total STAT3	Mono-	1:50 for IP	Abcam (Cambridge, UK)
pY-STAT3	p-STAT3 at Tyr 705	Mono-	1:500 for WB	Cell Signaling (Boston, MA)
pS-STAT3	p-STAT3 at Ser 727	Mono-	1:1000 for WB	Cell Signaling (Boston, MA)
STAT1	Total STAT1	Mono-	1:100 for IF<br>1:1000 for WB	Abcam (Cambridge, UK)
STAT1	Total STAT1	Poly-	1:50 for IP	Millpore(Deutschland, Germany)
pY-STAT1	p-STAT1 at Tyr 701	Mono-	1:500 for WB	Cell Signaling (Boston, MA)
pS-STAT1	p-STAT1 at Ser 727	Mono-	1:500 for WB	Cell Signaling (Boston, MA)
Ace- Lysine	acetylated proteins	Mono-	1:1000 for WB	Cell Signaling (Boston, MA)
GluN1	Total NMDAR1	Mono-	1:1000 for WB	Abcam (Cambridge, UK)
GluN2A	NMDAR2A C-term	Poly-	1:1000 for WB	Abcam (Cambridge, UK)
GluN2B	NMDAR2B C-term	Poly-	1:1000 for WB	Abcam (Cambridge, UK)
GluR1	Total GluR1	Mono-	1:500 for WB	Cell Signaling (Boston, MA)
GluR2	Total GluR2	Mono-	1:500 for WB	Cell Signaling (Boston, MA)
SYN	Total Synaptophysin	Poly-	1:1000 for WB	Abcam (Cambridge, UK)
SYT	Total Synaptotagmin	Mono-	1:1000 for WB	Abcam (Cambridge, UK)
P300	Total p300	Mono-	1:1000 for WB	Cell Signaling (Boston, MA)
PCAF	Human PCAF	Poly-	1:500 for WB	Abcam (Cambridge, UK)
GAPDH	Total GAPDH	Mono-	1:1000 for WB	Abcam (Cambridge, UK)
β-Actin	Human Actin	Poly-	1:2000 for WB	Abcam (Cambridge, UK)
Lamin B1	Nuclear Envelope Marker	Poly-	1:1000 for WB	Abcam (Cambridge, UK)
TAU5	Total tau	Mono-	1:1000 for WB	Abcam (Cambridge, UK)
HT7	Total human tau	Mono-	1:1000 for WB<br>1:200 for IF	Thermo Fisher (Waltham, MA)
AT8	Human PHF-tau	Mono-	1:200 for IF	Thermo Fisher (Waltham, MA)
pT205	p-Tau at Thr205	Poly-	1:1000 for WB	Signalway Antibody (College Park, Maryland)
pT231	p-Tau at Thr231	Poly-	1:1000 for WB	Signalway Antibody (College Park, Maryland)
pS396	p-Tau at Ser396	Poly-	1:1000 for WB	Signalway Antibody (College Park, Maryland)
pS404	p-Tau at Ser404	Poly-	1:1000 for WB	Signalway Antibody (College Park, Maryland)

Table S2 Human brain tissues used in the study

Case Number	Primary Neuropathologic Diagnosis	Secondary Neuropathologic Diagnosis	Formalin Tissue	PMI (hr)	Age at Death	Duration	ApoE	Race/Sex
E04-186	AD		√	7	72	13	E3/4	wf
E10-110	AD		√	42	47	17	E3/3	bm
E07-69	AD		√	~6	58	5	E3/4	wf
E05-87	AD		√	4	61	9	E3/4	wm
E06-155	AD		√	6.5	67	11	E2/3	wm
E05-74	Control		√	6	59		E2/3	bm
E05-130	Control	Diffuse plaques	√	3	52		E3/4	wf
E08-101	Control	Large cerebral hemorrhage	√	11.5	78		E3/3	wf
E11-33	Control	Microinfarct-pR	√	15	43		E3/3	bf
E04-32	Control	Microinfarct	√		70		E2/3	wm

Table S3 Primers used in the study

SYN	Forward	5’-TCCTCGCTGTCTAACGC-3’
	Reverse	5’-CATGGATCTTCTTCCCTTT-3’
SYT	Forward	5’-GAGGAAAGAACGCCATTA-3’
	Reverse	5’-GGGCACCTTGAAAGTAAA-3’
GluR1	Forward	5’- AGGTGCGGTTGTGG-3’
	Reverse	5’- CTCGGCGGCTGTAT-3’
GluR2	Forward	5’-ACGGCGTGTAATCC-3’
	Reverse	5’- TGTGCTCCAGGGTAT-3’
GluN1	Forward	5’-GTCCACCAGACTAAAGA-3’
	Reverse	5’-TCCCATCATTCCGT-3’
GluN2A	Forward	5’-CTTTTGAGGACGCC-3’
	Reverse	5’- AAATGAGACCCGATG-3’
GluN2B	Forward	5’-GGCTGACTGGCTACG-3’
	Reverse	5’- CTTGGGCTCAGGGAT-3’
β-actin	Forward	5’-CAAATGTTGCTTGTCTGGTG-3’
	Reverse	5’-GTCAGTCGAGTGCACAGTTT-3’
